# Case report: Early onset Marin-Amat syndrome after receiving ChAdOx1 nCoV-19 vaccination

**DOI:** 10.3389/fmed.2022.894755

**Published:** 2022-11-04

**Authors:** Ping-Feng Tsai, Ying-Jen Chen

**Affiliations:** ^1^Department of Medical Education, Taipei Veteran General Hospital, Taipei, Taiwan; ^2^Department of Ophthalmology, Tri-Service General Hospital, Taipei, Taiwan; ^3^School of Medicine, National Defense Medical Center, Taipei, Taiwan

**Keywords:** ChAdOx1 nCoV-19, vaccine, Bell's palsy, facial synkinesis, AstraZeneca

## Abstract

While vaccination against COVID-19 is still ongoing, some rare adverse events temporally related to vaccinations have been reported, particularly with ChAdOx1 nCoV-19. Here, a 77-year-old male presented to our outpatient department with persistent ptosis of his left eye for 1 month. He initially received vaccination with ChAdOx1 nCoV-19 and developed symptoms of Bell's palsy 3 days later. He received a 14-day course of prednisolone, but the ptosis persisted. Marin-Amat syndrome was compatible with his symptoms of ptosis exacerbation during orbicularis oris exertion. A temporal correlation between ChAdOx1 nCoV-19 vaccination and Bell's palsy without infectious or autoimmune diseases was delineated. Further studies are needed to clarify the possible relationship between these two events.

## Introduction

The coronavirus disease 2019 (COVID-19) global pandemic started in late 2019 and has recently been getting under control owing to the development of COVID-19 vaccines. One COVID-19 vaccine, ChAdOx1 nCoV-19, is an adenovirus-vectored vaccine and is nearly 100% effective in preventing serious events following COVID-19 infection (1). There are several commonly known side effects, including muscle pain, headache, and fever (1). However, there were no reports of Bell's palsy after ChAdOx1 nCoV-19 vaccination. Here, we report a case where the patient further developed a rare complication, Marin-Amat syndrome.

## Case description

**Figure 1:**
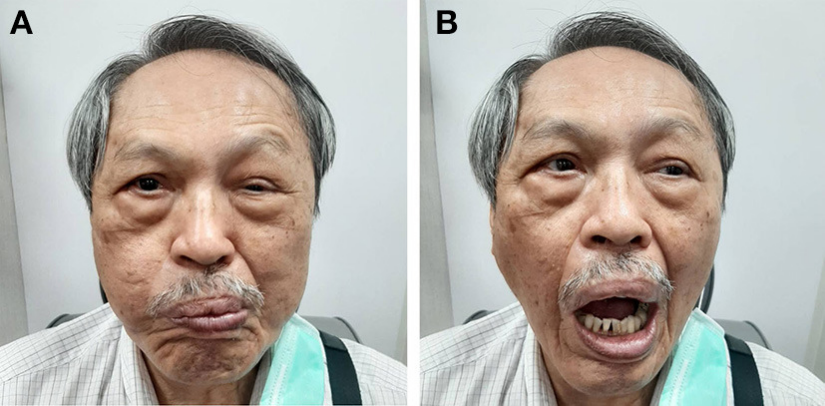
**(A)** This patient demonstrated puffy eyelid along with decreased lid height when his orbicularis oris contracted **(B)** Lid height was restored when his orbicularis oris relaxed.

**Figure 2:**
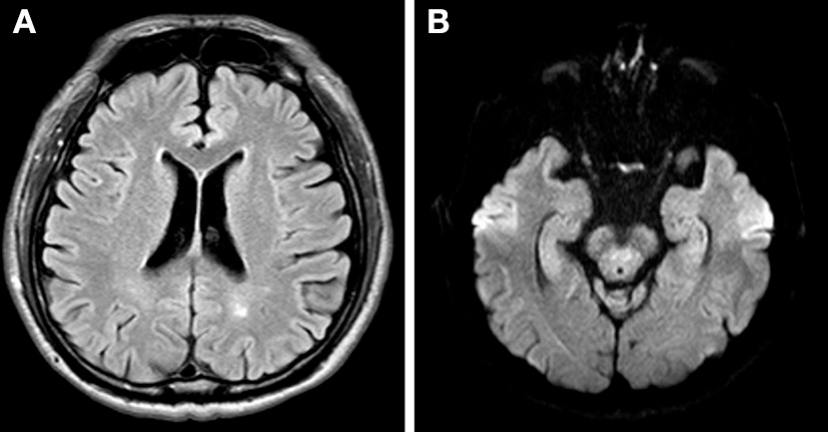
**(A)** Axial view of T2 MRI at lateral ventricle slice showed no obvious inflammatory lesion along frontoparietal cortex **(B)** Axial view of T2 MRI at the midbrain slice showed no obvious inflammatory change at oculomotor nerve.

A 77-year-old man was referred to our clinic complaining of a left droopy eyelid. His medical history included hypertension, chronic obstructive pulmonary disease, and hyperlipidemia, which was followed at a local medical clinic. He initially received the first dose of ChAdOx1 nCoV-19 (AstraZeneca, AZ) and started noticing that his left eyelid gradually became ptotic 3 days later. Eleven days after the initial vaccination, he experienced weakness of the left facial muscle. Physical examination revealed left-sided blink reflex impairment, ptosis, and weakness of left side facial muscle including orbicularis oris. Extraocular movement and light reflex were intact on both eyes. There was no vesicle/blistering noted on his face and his extraocular movement as well as light reflex were intact on both sides. Lab tests including thyroid function, vitamin B_12_, folate, glucose, cell count, electrolytes, renal function, liver function were within normal limits. Bell's palsy of his left side was diagnosed at our neurology clinic. He started prednisolone (40 mg QD) for 14 days; however, the left droopy eyelid showed little improvement. He then visited our ophthalmology clinic for persistent droopy eyelid despite a course of two-week prednisolone. His corrected visual acuity was 6/7.5 and 6/6 on his right and left eye, respectively. The intraocular pressure of both eyes was 18 mmHg. Slit-lamp examination was unremarkable except for puffy eyelids of his left eye with intermittent myokymic motion of his left upper and lower lids. Marginal reflex distance 1 (MRD1) of his left eye was 1 mm, in contrast to 3 mm on the right. His left eye was closed tightly as we asked him to open his mouth or smile (Figure 1). Note that his smile was symmetric at examination. Fundus imaging and optical coherence tomography of his macula and disc were unremarkable, and no obvious inflammatory lesions, including on the oculomotor and facial nerve region, were observed on the brain MRI (Figure 2). Marin-Amat syndrome was diagnosed.

## Discussion

This paper reported a case of Bell's palsy in a patient who received the ChA-dOx1 nCoV-19 vaccine. The diagnosis of Bell's palsy presented after excluding the known etiology resulting in facial palsy. Although few studies have reported facial weakness after ChAdOx1 nCoV-19 vaccination, none have reported idiopathic Bell's palsy after ChAdOx1 nCoV-19 vaccination, to our knowledge. Diogo et al. (2) reported a patient with facial weakness with geniculate ganglion enhancement on brain MRI after the first vaccination of ChAdOx1 nCoV-19 9 days later, along with symptoms of otalgia, implying Ramsey Hunt syndrome. Nicola et al. (3) reported a patient with facial palsy along with paraesthesia of the four limbs 10 days after the first vaccination. Electrophysiological findings were compatible with demyelinating motor polyneuropathy, which was diagnosed as Guillain Barre syndrome; the facial palsy was thought to stem from it (Table 1). In our case, a brain MRI revealed no obvious inflammatory changes or structural lesions compressing the facial nerve and oculomotor nerve, no obvious facial pain, vesicles, or other focal neurologic signs. However, the patient experienced an episode 3 days after vaccination, and symptoms culminated 11 days after vaccination; hence, the diagnosis of Bell's palsy.

**Table 1:** Demonstrates possible facial palsy etiologies following ChAdOx1 nCoV-19 vaccination.

**Case**	**Age/Sex**	**Time interval after AZ vaccination**	**Diagnosis**	**Treatment**	**Ref**
1	77/M	3	Bell's palsy	Prednisolone 40mg/day for 14 days	NA
2	42/M	9	Ramsey Hunt syndrome	Prednisolone 60mg/day for 7 days	(2)
3	59/M	10	Guillain Barre syndrome	IVIG 0.4 mg/kg for 5 days	(3)

Case 1 in this table was the case we presented, while case 2 and 3 were cases reported previously. Note that the etiology of facial palsy following ChAdOx1 nCoV-19 vaccination was completely different which resulted in different treatment and a different clinical course.

The etiology of Bell's palsy is speculated to be multifactorial. Virus reactivation and inflammation may play a major role in the pathogenesis of Bell's palsy (4). However, the relationship between facial palsy and COVID-19 vaccination remains largely unclear. Cases of facial palsy after COVID-19 vaccination are seen more commonly in those who receive mRNA vaccinations, particularly with the BNT162b2 vaccine. A case-controlled study (5) conducted in Israel enrolled 37 patients with acute onset facial palsy after exposure to the BNT162b2 vaccine. They compared these patients with 74 participants in the control group who were matched with the date of admission to eliminate bias owing to the different prevalence of vaccination and different seasonal incidence of Bell's palsy. They described the time interval between the first vaccination to facial palsy ranged from 3 to 14 days, and yet no statistically significant increase in odds ratio was found after BNT162b2 vaccination. Another study set to compare the incidence of facial palsy after mRNA vaccination was similar, even slightly lower, compared to those after receiving influenza or other viral vaccines (6). ChAdOx1 nCoV-19 was differed from other mRNA COVID-19 vaccines in its excipient, drug vector, and, as expected, its pharmacovigilance profile. Given that most of the studies were conducted to explore the association between mRNA COVID-19 vaccine and facial palsy, it remains largely unknown about the association between facial palsy and other COVID-19 vaccines, particularly ChAdOx1 nCoV-19.

Several disproportionality analyses were carried out *via* exploiting the information in the WHO pharmacovigilance database, VigiBase. Notably, cerebral venous thrombosis was found to have a high association with COVID-19 vaccines (7). COVID-19 vaccines were also found to be potentially associated with CNS demyelinating diseases, although a low association and was comparable with that of other viral vaccines (8). Our case highlighted a possible relationship between facial palsy and ChAdOx1 nCoV-19. Collectively, they demonstrate that COVID-19 vaccines may potentially lead to neurologic adverse events. The relationship between facial palsy and COVID-19 vaccines remained debatable, further epidemiological studies are warranted to clarify these observations.

This patient received a course of oral prednisolone and still experienced ptosis. Orbicularis oculi myokymia and synkinesis of the eyelid along with jaw motion were also observed, which is the key feature of Marin-Amat syndrome. Marin-Amat syndrome is a rare form of acquired facial synkinesis that manifests as involuntary eyelid closure with jaw movements. Ptosis is the most common complaint in these patients, followed by ptosis when eating. It is an overlooked etiology of ptosis and was traditionally thought as a late complication of the initial facial nerve injury. It is thought to result from aberrant facial regeneration (AFR) after an initial insult to the facial nerve (9). The pathogenesis of AFR is largely unknown, Celik et al. (10) demonstrated that the incidence of AFR following facial injury was related to the severity of injury and implied that the development of synkinesis would take place much earlier than 4 months. It is an imperative diagnosis to recognize in patients with persistent ptosis after initial facial injury.

Whether the aberrant facial regeneration is related to vaccination is unknown. The diagnosis of Marin-Amat syndrome is often overseen, and prudent evaluation for patients with ptosis, especially those with persistent ptosis after facial nerve injury, is needed. This case exhibits features of Marin-Amat syndrome, including intermittent orbicularis oculi myokymia, which was exacerbated by jaw movement. This feature implies a previous facial nerve insult, which is thought to be Bell's palsy, in our case.

## Conclusion

This is the first case report delineating an episode of Bell's palsy complicated with Marin-Amat syndrome following ChAdOx1 nCoV-19 vaccination. Further studies are warranted to clarify the relationship between Bell's palsy and ChAdOx1 nCoV-19 vaccination.

## Data availability statement

The original contributions presented in the study are included in the article/supplementary material, further inquiries can be directed to the corresponding author/s.

## Ethics statement

The studies involving human participants were reviewed and approved by Institutional Review Board of Tri-Service General Hospital. The patients/participants provided their written informed consent to participate in this study. Written informed consent was obtained from the individual(s) for the publication of any potentially identifiable images or data included in this article.

## Author contributions

Conceptualization: P-FT and Y-JC. Methodology, software, validation, formal analysis, investigation, resources, writing—review and editing, visualization, supervision, and project administration: Y-JC. Data curation and writing—original draft preparation: P-FT. All authors have read and agreed to the published version of the manuscript.

## Conflict of interest

The authors declare that the research was conducted in the absence of any commercial or financial relationships that could be construed as a potential conflict of interest.

## Publisher's note

All claims expressed in this article are solely those of the authors and do not necessarily represent those of their affiliated organizations, or those of the publisher, the editors and the reviewers. Any product that may be evaluated in this article, or claim that may be made by its manufacturer, is not guaranteed or endorsed by the publisher.
